# The importance of public participation in framing air pollution policy: outcome of a judicial review in New Delhi, India

**DOI:** 10.5588/pha.23.0047

**Published:** 2023-12-07

**Authors:** R. Singh, A. Frank

**Affiliations:** 1 Department of Architecture, School of Planning and Architecture, New Delhi; 2 Centre for Built Environment Policy, Information Sharing and Analysis Centre, Delhi; 3 Built Environment and Public Health Fellowship Programme, Tathatara Foundation, Bobbili, India; 4 Public Health, (Environmental and Occupational Health), Dornsife School of Public Health, Drexel University, Philadelphia, PA, USA

**Keywords:** environmental legislation, public participation, India, policy interventions, ambient air pollution

## Abstract

**SETTING:**

Air pollution, including particulate matter, causes health problems for residents of major cities around the world, including New Delhi, India. Public participation is important in framing policies related to such public health issues.

**OBJECTIVE:**

To study how the public’s comments on air pollution, which had been collected on the orders of the Indian Supreme Court, influenced air pollution policy in New Delhi.

**DESIGN:**

We filed a Right to Information Act, 2005 application with the Commission for Air Quality Management (CAQM) to obtain a copy of the comments. These were compiled and compared with key major areas identified in the policy released by the regulatory authority.

**RESULTS:**

There were a wide range of comments from experts, residents and resident associations. In total, 115 comments were compiled and studied, and several recurring themes were found to have been incorporated into the policy. This included the need to switch to green public transport and cycling, the use of alternative fuels and reduced agricultural crop residue burning.

**CONCLUSION:**

Our study revealed that the public and experts have indeed influenced the CAQM air pollution policy. This is important, as it highlights a democratic, inclusive and stakeholder-based approach. Nonetheless, a future concern lies in how this policy is translated into actionable regulations with effective implementation in the field.

Like many other cities in the world, New Delhi, India, has an air quality crisis and related public health issues due to the presence of high concentrations of particulate matter with a diameter of 2.5 µm (PM 2.5).^1^,^2^ The Lancet Commission on Pollution and Health reported that pollution was responsible for 9 million premature deaths in 2015, which makes it the largest cause of environmental risk leading to disease.^3^ The most common is respiratory illness: air pollutants, most notably particulate matter, cause asthma, chronic obstructive pulmonary disease (COPD), pneumoconiosis, hypersensitivity pneumonitis and lung cancer.^4^ There is also increasing evidence on the effects of ambient air pollution on cardiovascular diseases, with clinical manifestations including cardiac arrest, heart failure, stroke, ischaemic heart disease and myocardial infarction.^5^ There is also evidence of a relationship between air pollution and autoimmune diseases, dementia and cognitive decline,^6^,^7^ and it may also be a cause of fertility issues,^8^ with further links to other types of cancer.^9^ The issue with ambient pollution is that it can infiltrate indoor environments (where indoor pollutants are also being generated), further elevating pollution levels. Isolating the interior does not solve the problem, as there is a need for fresh air, which in many cases necessitates the use of external polluted air.^10^–^14^ Therefore, even indoor air quality depends in large part on solving ambient air quality problems through source control. For the benefit of human health, there must be better control of ambient air pollution through a scientific and multi-stakeholder approach, especially in large cities with high-density populations. This is also in line with the United Nations Sustainable Development Goals or SDG 3.9 on Health and Well Being and SDG 11.6 on Sustainable Cities.^15^,^16^ For example, China has undertaken measures (particularly in the context of international events such as the Beijing Olympics in 2008 and the Winter Olympics in 2022), to mitigate ambient air pollution at the venues.^17^ These measures included suspending production from polluting industrial enterprises, restricting motor vehicles, extensive monitoring and strictly controlling the dust released during construction.^18^ New Delhi is a large metropolitan area (the second largest after Tokyo), and has faced acute air pollution issues for decades, which the government is trying to address.^19^

The aim of this paper was to examine the comments from stakeholders, as requested by the Indian Supreme Court’s directive on air pollution in Delhi. This has enabled us to gain a better understanding of how stakeholder views have influenced air pollution policy.

## DESIGN

To obtain the public and expert stakeholder responses to the pollution control measures, we filed a Right to Information Act, 2005 application before the Commission for Air Quality Management (CAQM) for the National Capital Region and Adjoining Areas created under the Air Quality Management in National Capital Region and Adjoining Areas Act, 2021.^20^,^21^ A detailed analysis of the responses was performed and these were categorised, first subject wise and then into the most commonly recurring themes, and the number of occurrences of responses was collated. Other CAQM documents that led to the creation of the policy were also requested and key issues have been raised in the Discussion section. As this work uses data available in the public domain and does not involve human participant data, it is exempt from ethics review.

## RESULTS

There were some important initial observations. Of the 115 individual responses, 25 were from identifiable experts in the field based on their designation (i.e., their title or affiliation). Furthermore, three responses were from either a resident welfare association representative or part of a campaign group representing people directly affected by air pollution in New Delhi, or a group affected by government action in curbing pollution (e.g., an industry association that had a grievance regarding the compulsory use of piped natural gas). The number of recurring responses is shown in Table 1 and includes green transport/switching to public transport and cycling, followed by the related matter of switching to green or alternate fuel. As far as repetition by the public and experts was concerned, the familiar topic of crop residue burning was only third in importance. Although there are limitations to this approach in measuring importance, our objective was to provide insight into the responses, as these were later integrated into the policy formulated by the CAQM.

**TABLE 1:** The most frequently recurring responses among the 115 suggestions that were received from the members of the public and experts, New Delhi, India

Major themes	Number of times the theme was repeated in the responses. (n = 115)
Green (cleaner fuel)/alternative energy (fossil to renewable) sources	33
Green transport (fossil fuel to electric vehicle)/switch to public transport/promote cycling	35
Traffic control through the alternate use of cars with odd and even number plates	19
Curb consumption of industrial sources, including power plants	21
Construction management	15
Crop residue burning/farming intervention	24
Citizen engagement	16
Waste management	23
Afforestation/reforestation	14
Monitoring enhancement and grievance redressal	18
Policy and legal intervention: implementation of the Graded Response Action Plan	19

## DISCUSSION

After analysing and consolidating the stakeholder approach results, the CAQM formulated a policy known as the ‘Policy to Curb Air Pollution in the National Capital Region.’^22^ This policy categorises the air pollution mitigation measures for each sector. Each chapter addresses a specific sector. This policy offers a strategic plan for various government agencies and authorities across different jurisdictions. This is particularly significant due to the involvement of numerous state governments, numerous government agencies, and the central government. This is in line with what has happened globally in terms of air pollution regulation. However the multi-sectorial and multi-agency involvement makes this policy somewhat unusual and an example for other similar countries to follow and implement. One of the challenges that India faces as a low-middle income economy undergoing significant industrialisation, is the conflict between strict environmental controls vs. the need for economic growth.^23^

The process of formulating an air pollution policy shares similarities with initiatives in other regions, such as Bradford, UK, where stakeholder input and public focus groups, along with document analysis,^24^ were incorporated. In India, an adversarial justice system prevails, and the progressive stance of constitutional courts allows any member of the public to initiate legal action in a court of law.^25^,^26^ This differs from systems in which the administration may itself conduct the initial investigation and decision-making. In the Indian system, the responsibility lies with the executive branch of the government to demonstrate that efforts are being made to combat air pollution. The stakeholder approach of inviting public and experts to comment is useful and as no stakeholder is excluded, each person can have a voice. This is particularly important for experts who may not have been part of government-instituted committees, but have relevant knowledge and experience of the topic, along with a willingness to contribute. These outside opinions may well broaden the scope of problem solving.

Some countries have notified or released (under legal authority) environmental standards under national laws or legislation, making them enforceable.^27^ The real test of enforceability lies in the action applied to offenders that exceed the levels stated in the ambient air quality standards. These standards are prescribed by government agencies and are usually created through statute. In India, the body responsible is the Central Pollution Control Board, which was created under legislative action through the Water (Prevention and Control of Pollution) Act of 1974 and which was further entrusted with additional powers and functions by the Air (Prevention and Control of Pollution) Act of 1981.^28^,^29^ In the United States, this role is usually played by the Environmental Protection Agency (EPA), which is a federal agency created by executive order.

We discuss here the policy-related outcomes that have been developed by the CAQM under two scenarios: 1) before the occurrence of high pollution exposure levels; and 2) once the pollutant exposure levels have exceeded the standards, and there is an immediate need to reduce pollution levels.

### 1. Before the occurrence of high pollution exposure levels

This is the policy created by the CAQM for the period before pollution occurs.^22^ The policy to curb air pollution in Delhi was guided by the following principles:

Firstly, clean air for all, good health, well-being and increased productivity. Secondly, action for clean air requires speed, scale and urgency. Thirdly, equitous, inclusive, affordable, innovative approaches. Fourthly, scientific, technical and behavioural solutions. Fifthly, protect the vulnerable from the pollution risk. Sixthly, multi-sector policy response and a systems-based approach and finally, air pollution management needs an air shed and a regional approach.

The sector-wise heads for the abatement of air pollution are listed in Table 2, reproduced from CAQM.^28^ The dust mitigation measures are from the Central Pollution Control Board guidelines.^29^ To note, the final policy created by the CAQM has largely incorporated the suggestions by the general public, as mentioned before in Table 1. The comparison is shown in Table 3. The one issue leading to a special policy focus was the abatement of pollution by restricting the bursting of firecrackers. This was part of the suggestions received from the public but was not a recurring theme and has the added benefit of reducing noise pollution.

**TABLE 2:** The sector-wise action plans for the abatement of air pollution according to Central Government Commission on Air Quality Management policy for New Delhi and adjoining areas, India.^22^

S. no	Brief description of plan for the reduction of air pollution
1	Strengthening air quality monitoring and source apportionment
2	Reducing industrial pollution
3	Reducing air pollution from diesel generators
4	Reducing air pollution from thermal power plants within 300 km of Delhi
5	Reducing air pollution from vehicles and the transport sector
6	Use of clean fuels and electric mobility
7	Reducing air pollution through effective public transportation services
8	Reducing air pollution through effective road traffic management
9	Reducing air pollution by controlling municipal solid waste burning
10	Management of construction and demolition activities to reduce dust^32^
11	Reducing air pollution caused by crop residue burning
12	Reducing air and dust pollution from roads and open areas
13	Reducing air pollution through greening/plantation programmes
14	Reducing air pollution due to fireworks

**TABLE 3:** Comparison between the recurring themes based on stakeholder suggestions and the heads covered in the CAQM policy.

S. No.	The most common and recurring issues based on stakeholder comments	Heading under which the CAQM policy covered the issue
1	Green (cleaner fuel)/alternate energy (fossil to renewable)	Clean fuels and electric mobility Reducing air pollution from vehicles and the transport sector Abatement of air pollution from diesel generators
2	Green transport (fossil fuel to electric vehicles)/switch to public transport/promote cycling	Clean fuels and electric mobility Reducing air pollution through effective public transportation services
3	Traffic control through the alternate use of cars with odd and even number plates	Reducing air pollution through effective road traffic management Reducing air and dust pollution from roads and open areas
4	Curb industrial sources, including power plants	Reducing industrial pollution Reducing air pollution from thermal power plants within 300 km of Delhi
5	Construction management	Management of construction and demolition activities to reduce dust
6	Crop residue burning/farming intervention	Reducing air pollution caused by crop residue burning
7	Citizen engagement	
8	Waste management	Reducing air pollution by controlling municipal solid waste burning
9	Afforestation/reforestation	Reducing air pollution through greening/plantation programmes
10	Monitoring enhancement and grievance redressal	Strengthening air quality monitoring and source apportionment
11	Policy and legal intervention: implementation of the Graded Response Action Plan	Not dealt under a separate heading, but included in the policy

CAQM = Central Government Commission on Air Quality Management.

It is important to note that the above policy remains to be implemented and made enforceable. The CAQM has powers provided by the Indian Parliament to create rules and has created some with respect to crop residue burning. For example, a grievance mechanism has been created under Section 14 of the CAQM Act 2021.^30^ Under Section 12 of the Act, the CAQM also has the power to initiate cases on its own without a complainant. Penalties can also be imposed under the Act and related legislation. It is vitally important that the policy be enforced by the executive wing of the government.

### 2. After pollutant exposure levels have exceeded standards

Another part of policy developed by CAQM is the need for redress after exposure levels have exceeded standards, called the Graded Response Action Plan (GRAP).^31^ This allows for urgent remedial steps to be taken to reduce air pollution in New Delhi and adjoining regions. This has an inbuilt disadvantage in that it requires pollution to reach excessive levels before remedial action can be initiated. To avoid this, the Supreme Court highlighted the need for a predictive system to warn of high pollution conditions so that pre-emptive action could occur. This may be based on meteorological and statistical modelling by domain experts. The GRAP for pollution in Delhi is based on a four-level system comprising Stages 1 to 4 (moderate to poor, very poor, severe and emergency). The revised GRAP has been provided in the GRAP notification.^31^

## CONCLUSION

This study set out to study the comments from members of the public and domain experts collected through open public invitation. These were categorised into recurring themes, with a primary focus on green transportation, transitioning to public transit, and cycling, closely followed by the related aspect of adopting green or alternative fuels. Crop residue burning ranked as the third most important concern. This led to the creation of the policy by the CAQM. The inclusion of stakeholder suggestions as per the suggestion of the Indian Supreme Court sets a precedent for other countries with problems that are similar to India: it is a democratic decision-making process for something that effects all our lives. The next challenge lies with the executive wing of the government and the CAQM to implement the policy effectively. Future research in this area should monitor implementation of the policy and its effects in reducing air pollution in New Delhi and the adjoining areas.
